# The potential applications of microparticles in the diagnosis, treatment, and prognosis of lung cancer

**DOI:** 10.1186/s12967-022-03599-x

**Published:** 2022-09-05

**Authors:** Yu Liu, Sufei Wang, Hui Xia, Xueyun Tan, Siwei Song, Shujing Zhang, Daquan Meng, Qing Chen, Yang Jin

**Affiliations:** 1 Physical Examination Center, Union Hospital, Tongji Medical College, Huazhong University of Science and Technology, Wuhan, 430022 China; 2 Department of Respiratory and Critical Care Medicine, NHC Key Laboratory of Pulmonary Diseases, Union Hospital, Tongji Medical College, Huazhong University of Science and Technology, 1277 Jiefang Avenue, Wuhan, 430022 Hubei China; 3 Department of Pediatrics, Union Hospital, Tongji Medical College, Huazhong University of Science and Technology, Wuhan, 430022 Hubei People’s Republic of China

**Keywords:** Microparticles, Lung cancer, Tumour microenvironment

## Abstract

Microparticles (MPs) are 100–1000 nm heterogeneous submicron membranous vesicles derived from various cell types that express surface proteins and antigenic profiles suggestive of their cellular origin. MPs contain a diverse array of bioactive chemicals and surface receptors, including lipids, nucleic acids, and proteins, which are essential for cell-to-cell communication. The tumour microenvironment (TME) is enriched with MPs that can directly affect tumour progression through their interactions with receptors. Liquid biopsy, a minimally invasive test, is a promising alternative to tissue biopsy for the early screening of lung cancer (LC). The diverse biomolecular information from MPs provides a number of potential biomarkers for LC risk assessment, early detection, diagnosis, prognosis, and surveillance. Remodelling the TME, which profoundly influences immunotherapy and clinical outcomes, is an emerging strategy to improve immunotherapy. Tumour-derived MPs can reverse drug resistance and are ideal candidates for the creation of innovative and effective cancer vaccines. This review described the biogenesis and components of MPs and further summarised their main isolation and quantification methods. More importantly, the review presented the clinical application of MPs as predictive biomarkers in cancer diagnosis and prognosis, their role as therapeutic drug carriers, particularly in anti-tumour drug resistance, and their utility as cancer vaccines. Finally, we discussed current challenges that could impede the clinical use of MPs and determined that further studies on the functional roles of MPs in LC are required.

## Background

Lung cancer (LC) has the highest morbidity and mortality among all types of cancers and accounts for the majority of cancer-related deaths worldwide [1, 2]. The overall 5-year survival rate of LC patients is less than 15% [3]. Currently, carcinoembryonic antigen, fragments of cytokeratin 19, neuron-specific enolase, and pro-gastrin-releasing peptides are the most common tumour markers used in the clinical diagnosis of LC. However, due to the limited sensitivity and specificity of these markers, most LC patients who are diagnosed at an advanced stage usually have a poor prognosis. In addition, despite significant advances in LC research and anticancer therapies, including surgery, radiotherapy, chemotherapy, molecular targeted therapies, and immunosuppressive agents [4], the overall survival rate of LC remains low [3]. Therefore, further studies on the molecular mechanisms, early detection, and targeted therapies of LC are vital.

**Fig. 1:**
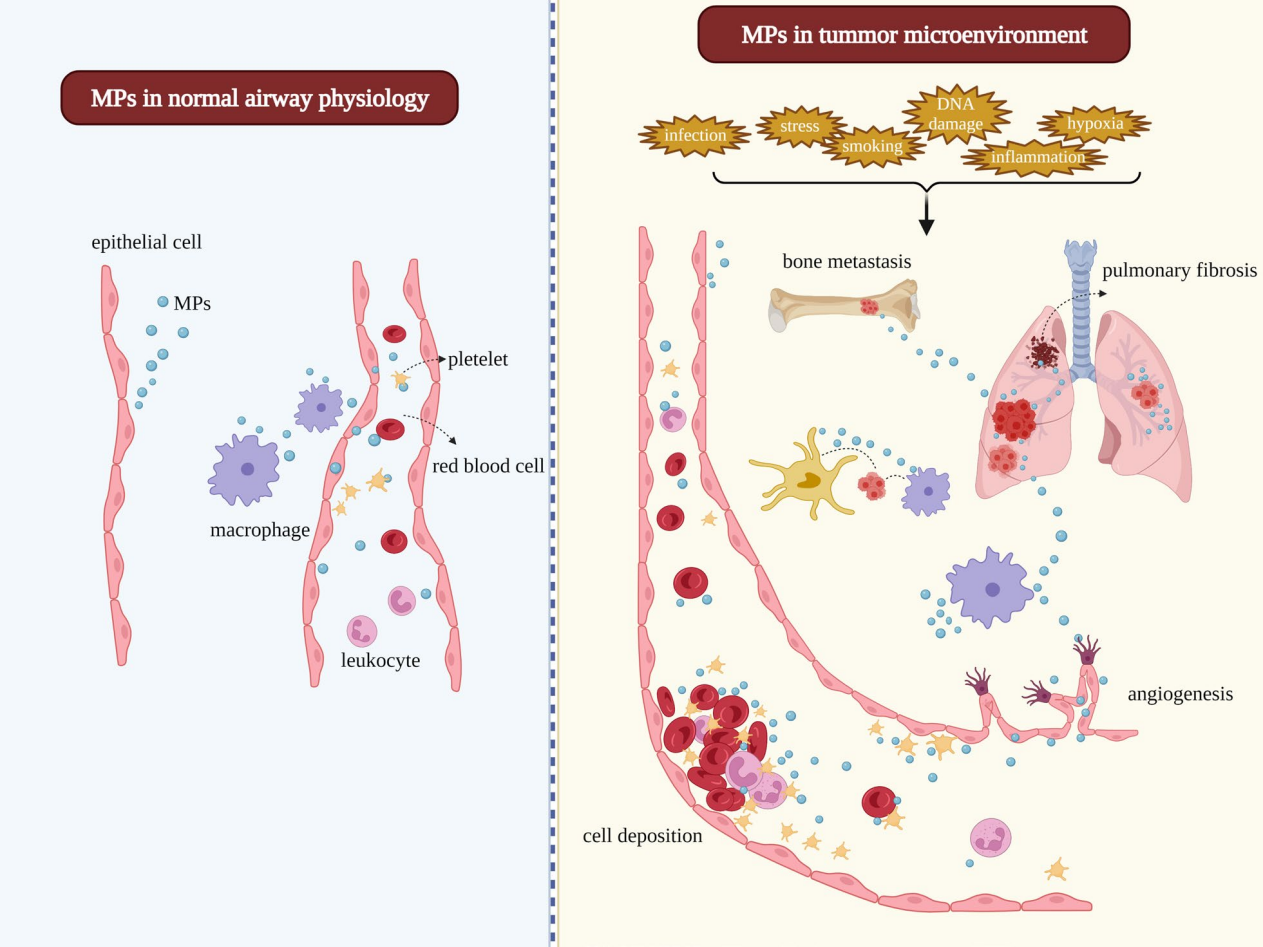
The promotion of lung cancer proliferation, invasion, and metastasis by microparticles

Extracellular vesicles (EVs) are composed of exosomes, microparticles (MPs), and apoptotic bodies (Table 1). These vesicles can be detected in the supernatants of cell cultures and in various biological fluids, such as blood, urine, sputum, breast milk, and synovial, bronchoalveolar lavage, pleural effusion, and ascites fluids [5]. MPs, also called microvesicles, shedding vesicles, or ectosomes, are released into the extracellular space from the surface membranes of cells [6]. In the LC microenvironment, MPs can be found in normal, tumour-infiltrating (e.g., activated platelets, monocytes, and lymphocytes), and cancer cells (Fig. 1). MPs are capable of transferring surface receptors from one cell to another and delivering proteins, mRNA, bioactive lipids, organelles (e.g., mitochondria), and even vaccines based on the delivery of tumour lysates into target cells [7–13]. MPs shed from various tumour cell lines or tumour cell-related lines have been thought to facilitate extracellular matrix invasion and evasion of the immune response [14], whereas those secreted by normal endothelial cells might exhibit protective effects [15]. Endothelial-derived microparticles (EMPs) enable cells to dispose of potentially harmful and redundant compounds, thereby promoting cellular survival [16–18]. Several recent studies have found that MPs may facilitate intercellular communication [19–22]. MPs have been proposed as indicators of progressive and aggressive LC. The basal values of circulating MPs can serve as an independent predictor of survival outcomes in advanced non-small cell LC (NSCLC) patients. Due to their capacity to pack large amounts of biological information, tumour-derived MPs (TMPs) are ideal candidates for delivering therapeutic agents to tumour cells and may play a crucial role in the development of novel and effective tumour vaccines. Further, TMPs loaded with anti-tumour drugs could reverse drug resistance.

**Table 1:** Identification of the subtypes of extracellular vesicles

EV subtypes	Exosomes	Microparticles
Alternative	Extracellular vesicles	Ectosomes or Microvesicles or Extracellular vesicles
Origin	Endosomal membrane	Plasma membrane
Form	Inward budding	Outward budding
Medium	Multivesicular endosomes (MVE)	None
Size	30–100 nm	100–1000 nm
Sedimentation	100,000×*g*	10,000×*g*
Detection	Electron microscopy, NTA, TRPS, Bead-based flow cytometry, Fluorescence-triggered flow cytometry	Conventional scatter-triggered flow cytometry
Mechanisms of the biogenesis	1. ESCRT dependent mechanism [146, 147] 2. Synthesis of ceramide that induces vesicle curvature and budding [148] 3. Tetraspanin-mediated organization of specific proteins such as the amyloidogenic protein and the premelanosome protein [149, 150]	1. Characterized by an increase in cytosolic calcium concentration [44] 2. Apoptosis-dependent microparticle formation mechanism [45]
Annexin V binding capacity	No/Low Annexin V binding capacity	High Annexin V binding capacity
Release	Constitutive and/or cellular activation	Cellular activation and early apoptosis

MPs induced by environmental cues (activation, injury, hypoxia, or apoptosis) are involved in cancer cell initiation, progression, and metastasis; extracellular matrix remodelling; multidrug resistance; and modulation of inflammation [23], thrombosis [24], endothelial dysfunction [25], tissue remodelling [26], angiogenesis [27], and immunological reactions [28]. The levels of circulating MPs are increasingly elevated in many types of cancers, including haematological malignancies [29, 30], breast cancer [31], ovarian cancer [32], and colorectal cancer [33]. Najjar et al. firstly found that increased circulating endothelial cells (CECs) and MPs during or after chemotherapy can act as predictive biomarkers of tumour progression in advanced NSCLC [34]. However, available data on the association between the levels of circulating MPs and LC are limited [35, 36]. In addition, most studies do not distinguish MPs from exosomes and excessively focus on the role of exosomes rather than that of MPs. Thus, this review solely focused on the biogenesis, components, isolation, quantification, and potential clinical implications of MPs in LC.

## The biogenesis, components, isolation, and quantification of microparticles

### Microparticles biogenesis

MPs were first described as “platelet-like activity” in 1955 and later as “platelet dust” in 1967. Multiple studies have investigated the composition, origin, and roles of these particles, leading to the gradual replacing of the name "platelet dust" with "microparticles" [37–39]. Almost all cell types are capable of producing and shedding MPs [40]. MPs are formed by the outward blebbing of the plasma membrane and subsequently released by the proteolytic cleavage of the cytoskeleton and expression of antigens specific to their parental cells [41]. Moreover, MPs contain many proteins and lipids similar to those found in the membranes of their parental cells and may also contain mRNA. Multiple mechanisms of MP biogenesis have been described; however, the two best-known mechanisms are cell activation and apoptosis [42]. Cell activation causes MP shedding, which starts within minutes of adding the right agonist and is characterised by higher calcium levels in the cytosol [43, 44]. Signs of damage (like injury, hypoxia, or apoptosis) cause the endoplasmic reticulum to release calcium into the cytosol. This causes the cytoskeleton to change shape and the phospholipid asymmetry to flip. When phosphatidylserine moves out of the cell, it causes the cell membrane to bulge outward, which results in a fissure. Consequently, MPs express both phosphatidylserine and surface proteins related to their parental cells on their outer membranes. In apoptosis-dependent MP formation, dynamic membrane blebbing occurs after cell contraction and DNA fragmentation, and it usually lasts for hours [45]. During membrane blebbing, the molecular regulators of MPs release cytosolic calcium, Rho kinases, GTPase, RhoA, mitogen-activated protein kinases, and nuclear factor-κB [46]. The mechanisms by which MPs develop and bud from cell plasma membranes are still largely unknown. Therefore, we must continue to gain more understanding on the underlying mechanisms that allow MPs to carry certain proteins, RNAs, and DNAs.

### Molecular components of microparticles

The Vesiclepedia database (www.microvesicles.org) [47] catalogues proteins, lipids, and acids identified in MPs from various sources. MPs contain a broad spectrum of bioactive substances and receptors on their surface, including lipids, nucleic acids, and proteins, that reflect not only their cellular origin but also the stimulus that triggered their biogenesis and secretion. MPs may shuttle these molecules between neighbouring cells via systemic transport or distant anatomic sites where they may induce signalling pathways or directly alter the phenotype of specific recipient cells. As mentioned above, the composition of MPs determines their role in cell communication.

### Proteins in microparticles

Many proteins, including selectins, integrins, cluster of differentiation (CD) 40, matrix metalloproteinases, phosphatidylserine, ADP-ribosylation factor 6, and Rho family members, have been indicated as MP-specific [48, 49]. One study identified 910 different proteins in salivary macrovesicles from healthy participants and patients with LC. In particular, 626 proteins were found in salivary MPs from patients with LC [50]. Among these, 243 proteins were identified as dysregulated candidates and 284 as unique to patients with LC, of which 40 were originally from distal organs or tissues, and nine originated from the lungs. In total, 109 proteins were upregulated and 134 were downregulated (Table 2).

**Table 2:** Salivary microparticle proteins in normal participants and patients with lung cancer [50]

Microparticles in the saliva	Proteins
In normal participants and patients with lung cancer	910
In patients with lung cancer alone	626
Unique proteins	284
Originated in distal organs or tissues	40
Originated in the lung	9
Upregulated proteins	109
Downregulated proteins	134

Proteins play a key role in LC progression. For example, Ras GTPase-activating-like protein 1 (IQGAP1) acts as a signal interrogator in LC cell proliferation. BPI fold-containing family A member 1 (BPIFA1) takes part in the innate immune response of NSCLC [51]. Cornulin is considered a survival factor related to apoptotic cell death and calcium release [52]. Mucin 1 is cross-processed and presented to antigen-specific CD8^+^ T cells when carried by MPs. Internalised and soluble mucin 1 is retained in the endolysosomal/HLA-II compartment and does not induce T cell response [53, 54]. Studies have shown that these proteins may be exploited for possible non-invasive detection of LC.

### Nucleic acids in microparticles

MicroRNAs (miRNAs) are indispensable for cell differentiation, proliferation, maturation, and apoptosis [55]. miRNAs (e.g., miR-21, miR-19, miR-133, miR-146, miR-126, and miR-223) are detectable in platelet-derived MPs (PMPs) [56]. In platelets and PMPs, miR-223 is the most abundant miRNA [57, 58]. The expression of miR-223 is aberrant in breast cancer, gastric cancer, LC, and ovarian cancer [59–62]. As such, it is considered to be a member of an emerging family of cancer-promoting miRNAs known as oncomiRs. miR-223 is also the most upregulated miRNA in recurrent tumours [62] because it directly targets the 3′ UTR of erythrocyte membrane protein band 4.1-like 3 (EPB41L3) [61]. PMPs can effectively deliver miR-223 into human LC cells via EPB41L3, promoting tumour invasion. miR-223 not only directly binds to the 3′ UTR of the EPB41L3 mRNA transcript, inhibiting EPB41L3 translation, but also decreases the cellular levels of the EPB41L3 protein. As such, increased motility and decreased adhesion are observed in LC cells, inducing tumour cell invasion. These MP-encapsulated miRNAs can be successfully transported into target cells to silence target genes, hence influencing recipient cell function [63, 64]. Therefore, cell-secreted miRNAs in MPs can serve as a novel class of signalling molecules to mediate intercellular communication from a distance. Some RNA transcripts found in cancer cell-derived MPs can function as messages or biomarkers that can be recognised using available technology or a very sensitive way.

### Methods for microparticles isolation and quantification

The Minimal Information for Studies of Extracellular Vesicles provides research guidelines for EVs to promote the transparency and reproducibility of EV studies [65]. Currently, multiple accepted methods, such as ultracentrifugation (including differential centrifugation [DU]), microfluidics, ultrafiltration, immunoaffinity chromatography, and size-exclusion chromatography, have been successfully used for the isolation of MPs [66]. Immunoaffinity chromatography cannot distinguish MPs from exosomes because exclusive markers for each one have not been identified yet. This method often serves as a purification method after isolating MPs from large sample volumes [67]. Traditional ultracentrifugation, described as the most dependable method, consists of a series of centrifugation cycles with varying centrifugal forces and durations to separate EVs based on their density and size differences [66]. DU is an ideal method of EV isolation for many laboratories due to its low-cost and high-throughput properties. Microfluidics is an appealing approach due to its fast and simple operation. Small-volume samples may even be used for disease diagnosis. Combination methods can improve the purity of the collected vesicles [68]. Size-exclusion chromatography can be followed by ultracentrifugation or ultrafiltration to concentrate isolated but diluted MPs [67]. Methods of separation are typically selected with a clinical goal.

Particle number can be measured by light-scattering technologies (e.g., nanoparticle tracking analysis [NTA]), standard flow cytometry (FCM) [69–72], tunable resistive pulse sensing (TRPS) [73], cryogenic electron microscopy [74], platform combining surface plasmon resonance with atomic force microscopy (AFM) [75], or dynamic light scattering (DLS). AFM can be used to study the size, antigenic properties, and number of defined subsets of MPs [76]. Single-particle analyses like NTA, TRPS, and DLS are now widely used to measure the number and size of EVs. However, they do not give enough information about phenotype and are not the best way to measure vesicles that are larger than 200 nm. Until recently, FCM could analyse only large EVs or the population of smaller EVs captured on beads prior to analysis [77]. FCM remains the most extensively used technique for the enumeration and characterisation of MPs [78, 79]. To achieve better specificity of EV subtype separation, two or more methods are used for EV characterisation. Taken together, essential technologies need to be investigated further to ensure the reliable isolation of disease-specific MPs from body fluid and tissue samples, as well as to rigorously discriminate these vesicles from those formed by non-diseased cells. Further, it is important to develop the necessary methods for high-sensitivity identification of specific cargo proteins, RNAs, or miRNAs.

## Different microparticles in the lung cancer microenvironment

Cells can release MPs derived from many sources, including leukocytes, platelets, erythrocytes, endothelial cells, macrophages, and tumour cells, at each stage of their lifecycle. In LC, MPs can play a role in inflammation, thrombus formation [24, 26, 29, 80], and angiogenesis [24, 29–31]. Furthermore, PMPs exhibit pro-angiogenic activity, which can promote capillary-like structure formation and pro-angiogenic factor production [14, 20, 23, 25]. Conversely, EMPs can be either pro- or anti-angiogenic, depending on exposure to factors stimulating their production [24].

Platelets release more MPs when various inflammatory factors are upregulated and under disease conditions, such as malignancy [81], sepsis [82], thrombocytopenia [83], arterial thrombosis [26], thrombotic thrombocytopenia [84], uraemia [85], and rheumatoid arthritis [86]. PMPs are activated in a calcium flux-calpain-dependent manner [87]. TMPs regulate tumour microenvironment (TME); increase tumour invasion, metastasis, and angiogenesis [88]; and even escape immune surveillance. In the airway, alveolar macrophages are a major source of bronchoalveolar lavage fluid cellular components and have a significant influence on inflammation. After interacting with different cells in a pathological state, macrophage-derived MPs (MMPs) are transported to various types of respiratory cells, such as lung epithelial cells, endothelial cells, fibroblasts, and monocytes, ultimately leading to cellular homeostasis and differentiation [89]. EMPs can carry a wide range of transcripts and have angiogenic activity mainly in quiescent endothelial cells by promoting endothelial cell proliferation, organising capillary-like structures, and preventing apoptosis. Elevated levels of circulating lymphocyte-derived MPs (LMPs) are associated with disease progression in advanced NSCLC [90]. The total MPs, PMPs, and LMPs increased significantly with disease progression in patients with advanced NSCLC who were treated with immune checkpoint inhibitors. The participation of different MPs in the key steps of cancer progression through different functions has been considered. The surface antigens that characterise and used to enumerate the functions of different MPs are summarised in Table 3.

**Table 3:** List of different cellular surface markers according to origin and function

Type	Markers	Antigen	Cellular origin	Function	Refs.
PMPs					
	CD41	αIIb chain	Platelet	They can bind together to form the glycoprotein GPIIa/IIb (integrin αIIbβ3) which is a member of the integrin transmembrane family. The major binding site contains the arginine-glycine-aspartic acid (RGD) sequence presenting in several adhesive proteins, such as von Willebrand factor (VWF). Inside-out signalling activates the complex, permitting binding to VWF through platelet activation	[151]
	CD61	β3 integrin			
	CD42a	GPIb/V/IX	Platelet	Two membrane glycoproteins that bind together to form (GP)Ib‐IX‐V complex. (GP)Ib‐IX‐V is expressed on platelets' surfaces and is involved in thrombosis, acting as a receptor for vWF and other molecules such as thrombin	[152]
	CD42b	GP1bα			
	CD62P	P‐selectin	Platelet	Also known as Platelet Activation‐Dependent Granule to External Membrane Protein (PADGEM) or Granule Membrane Protein 140 (GMP‐140). It is a transmembrane glycoprotein that is expressed by activated platelets and plays a key role in immune cell adhesion and rolling	[153]
	PAC1	GPIIb/IIIa	Platelet (activation marker)	It is present only on the surface of activated platelets and recognises an epitope on the GPIIb/IIIa complex of activated platelets at or near the platelet fibrinogen receptor	[154]
	CD63		Platelet	CD63 is located in the lysosomal integral membrane and is rapidly redistributed to the platelet surface when platelets are stimulated	[35]
	CD40L		Platelet	It can act as a good candidate for platelet activation in an auto-amplification loop. CD40L is involved in inflammation and a panoply of immune-related and vascular pathologies	[155]
EMPs					
	CD54	Intercellular adhesion molecule (ICAM‐1)	Endothelial cell	It is an inducible cell adhesion protein that plays a role in leukocyte and endothelium interaction to regulate vascular permeability. It is also induced by inflammation and is expressed on a wide range of immune cells such as monocytes and macrophages	[156]
	CD62E	Endothelial leukocyte adhesion molecule 1(E‐selectin/ELAM-1)	Endothelial cell (activation marker)	A cell adhesion molecule is induced in response to inflammation and is thought to play a role in recruiting leukocytes to the sites of injury	[154, 157, 158]
	CD105	Endoglin	Endothelial cell	CD105 is a component of the receptor complex of Transforming Growth Factor (TGF)-βinvolved in cellular proliferation, differentiation and migration	[158–162]
	CD144	Vascular endothelial cadherin (VE‐cadherin)	Endothelial cell	Constitutively expressed at endothelial adherence junctions. It plays a role in controlling vascular permeability and leukocyte extravasation	[163]
	CD31	Platelet and endothelial cell adhesion molecule (PECAM‐1)	Endothelial cell	It is expressed in most vascular compartment cells. It is found at cell junctions in endothelial cells and plays various roles in inflammation and vascular biology	[164]
	CD146	Melanoma cell adhesion molecule (MCAM)	Endothelial cell	An adhesion molecule involved in cell signalling, vascular permeability, and immune response	[165]
	CD106	Vascular cell adhesion molecule (VCAM‐1)	Endothelial cell	It is a transmembrane glycoprotein and is a marker of endothelial cell activation and inflammation	[166]
	CD51	Vitronectin receptor/vitronectin and fibronectin receptor	Endothelial cell	It may be related to increased airway inflammation and repair processes in response to injury	[167]
TMPs					
	CD47		Tumour cell	CD47 interacts with signal-regulatory protein alpha (SIRPα) on macrophages and monocytes to prevent phagocytosis	[168]
	EpCAM		Tumour cell	It promotes tumour invasion when expressed in its highly-glycosylated isoform on tumour-derived MPs (T-MPs) [2]	[88, 169, 170]
	CD147	Extra-cellular matrix metalloproteinase inducer (EMMPRIN)	Tumour cell	T-MPs stimulate cancer cell invasion via a direct feedback mechanism dependent on highly glycosylated EMMPRIN by activation of the p38/MAPK signalling pathway	[88]
MMPs					
	CD11b		Monocyte	It may participate in degrading alveolar walls	[88]
	CD11c				
	CD14	Lipopolysaccharide receptor (LPS-R)	Monocyte	lPs receptor, present on the surface of monocytes/macrophages	[154]
	CD64		Macrophages	Alveolar macrophage surface marker	[171]
	CD16		Macrophages	Act as a surface marker of macrophage activation	[172]
	CD32	FcyRII	Macrophages	It plays a major role in the regulation of humoral immune responses	[173]
LMPs					
	CD13	Aminopeptidase N	Leukocyte	Present on the surface of granulocytes and monocytes	[154]
	CD56	Neural cell adhesion molecule (NCAM)	Leukocyte	It plays an important role in cell–cell adhesion	[154]
	CD45		Leukocyte	Pan leukocyte marker	[154]

## Clinical applications of microparticles for diagnosis, prognosis, and therapy

Liquid biopsy, a minimally invasive test, is a promising alternative to tissue biopsy for the early screening of LC [90, 91]. MPs can be found in blood, urine, sputum, breast milk, synovial, and bronchoalveolar lavage. For high stability, biological fluids can be regarded as ideal materials for liquid biopsies. The composition of MPs mirrors the contents of donor cells and bears the hallmarks of the regulated sorting mechanisms of these cells, providing diagnostic utility for LC. The diverse biomolecular information from MPs, including that on proteins, lipids, various metabolites, and nucleic acids, provides prospective biomarkers for LC risk assessment, early detection, diagnosis, prognosis, and surveillance.

### Microparticles as diagnostic biomarkers for lung cancer

Profiling proteomics has revealed a variety of EV-associated protein cargoes, including receptors, transcription factors, enzymes, signalling proteins, lipid raft proteins, cytoskeletal and extracellular proteins, vesicle-trafficking proteins, and immune-interacting proteins [92, 93]. BPIFA1, Mucin 5B, and Ras GTPase-activating-like protein can prove useful as non-invasive biomarkers of LC [50]. Moreover, SPARC-like protein 1 (SPARCL1), IQGAP1, BPIFA1, and cornulin are potential candidate proteins abnormally expressed in multiple types of cancers, especially LC. SPARCL1 is classified as a member of a larger family of secreted acidic and cysteine-rich matricellular proteins [94]. According to Isler et al., SPARCL1 is downregulated in human NSCLC and thus can be effectively identified as a predictive factor. A survey suggested that SPARCL1 downregulation is mediated by transacting factors that bind to its exon 1 [95]. IQGAP1 participates in multiple cellular actions (i.e., transcription, cell–cell adhesion, and cytoskeleton regulation) by targeting calmodulin, cell division control protein 42, Ras-related C3 botulinum toxin substrate 1, actin, β-catenin, and E-cadherin. BPIFA1 predominantly exists in the upper respiratory tract and salivary glands of both mice and humans and participates in the lung immune response. Cornulin is a newly discovered member of the “fused gene” family and the product of the novel gene *c1orf10*, an oesophageal-specific and cancer-associated gene located on 1q21. The *c1orf10* gene encodes a Ca^2+^-binding protein in the upper layer of squamous epithelia that plays an important role in epidermal differentiation and is a marker of late epidermal differentiation.

### Microparticles as prognostic biomarkers for lung cancer

MPs have been proposed as indicators of progression and aggressiveness of NSCLC [96]. For example, the level of EMPs is a useful diagnostic marker for LC [97]. The basal value of circulating MPs serves as an independent predictor of 1-year clinical outcomes in patients with advanced NSCLC [98]. A level of circulating EMPs ≥ 1100.5 count/mL is one of the most important predictors of 1-year mortality in patients with end-stage NSCLC, with sensitivity and specificity rates of 77.6% and 56.9%, respectively. In addition, patients with small-cell LC who initially responded to chemotherapy exhibited low basal MP numbers.

EMPs activate matrix metalloproteases, which are involved in the degradation of the extracellular matrix and the release of growth factors that are essential for tissue remodelling, angiogenesis, and metastasis [99]. Moreover, Tseng et al. found that circulating EMPs are more closely associated with small cell carcinoma than squamous cell carcinoma [36]. Squamous cell carcinoma tends to have a slower growth rate and spread later in the course of the disease than small cell carcinoma and adenocarcinoma [100]. As a result, squamous cell carcinoma displays a slower rate of metastasis and lower degree of angiogenesis in the host microenvironment than other types of LC, leading to a lower level of EMPs.

Najjar et al. found that before chemotherapy, the total MPs in patients with stage IV NSCLC are significantly higher than those in patients with stage III NSCLC. Further, the rate of change in total MPs after chemotherapy can predict disease progression [34]. Elevated levels of circulating LMPs are associated with disease progression in advanced NSCLC [90]. According to this study, total MPs, PMPs, and LMPs increased significantly with disease progression in advanced NSCLC with treatment. Due to their significance as prospective lung cancer biomarkers and biological communication carriers, MPs have drawn the attention of the scientific community. MPs have the potential to be used as a specimen for liquid biopsy with a higher sensitivity and accuracy.

### Therapeutic applications of microparticles for lung cancer

#### Microparticles as a novel mode of drug delivery

Remodelling the TME, which profoundly influences immunotherapy and clinical outcomes [101, 102], is an emerging strategy to improve immunotherapy [103]. Due to their capacity to package large amounts of biological information, TMPs are ideal for delivering therapeutic agents (e.g., oncolytic adenoviruses, chemotherapeutic drugs, nucleic acids, antibodies, and antigens) to tumour cells, effectively killing the cancer cells [104–106]. Drug MPs can be directly injected into superficial solid tumours or delivered to target tumour cells through a drainage tube in cases of malignant pleural effusion and ascites. Drug MPs can also be used to target tumour-associated macrophages, key players in tumour immunosuppression, cancer stemness, and metastasis [107]. M1-like macrophages remodel the TME by reducing the number of immunosuppressive cells and augmenting T cell infiltration, thereby promoting effective antitumor T cell immunity [107]. Drug-packaging TMPs efficiently mobilise endogenous neutrophils and induce intrinsic antitumour activities. The attracted neutrophils display a mature CD11b^+^/CD15b^+^ phenotype and kill tumour cells by releasing reactive oxygen species and NO into the TME [108].

Autologous TMPs packaged with chemotherapeutic agents have been approved as a new biological therapy for malignant tumours due to their demonstrated safety and tolerability [106]. According to our previous studies, TMPs packed with methotrexate, a chemotherapeutic drug, markedly restrict the growth of malignant pleural effusion and provide a survival benefit in both animal and human experiments [109, 110]. Ran et al. found that TMPs can act effectively deliver oncolytic adenoviruses to tumours and induce highly efficient cytolysis [111]. Additionally, Chen et al. proposed a donor cell-assisted membrane biotinylation strategy to achieve biocompatible quantum dot labelling of TMPs, thereby creating a novel method for nanocarrier preparation [112]. MPs are nontoxic and stable in body fluids; however, their efficacy for drug delivery to target cells still requires more research before they can be exploited. Efforts should be made to load isolated MPs with specific therapeutic cargos (drugs, RNAs, or DNAs) and then employ them to effectively deliver therapy to diseased or injured target cells.

#### Microparticles and drug resistance

Therapeutic resistance is the leading cause of a poor prognosis for cancer. Progression of cancer is a complicated process dependent on interactions between the tumour and TME [113]. Although TMPs play important roles in promoting the formation of tumour drug resistance, increasing studies have focused on therapeutic applications of MPs to reverse drug resistance.

#### Drug resistance of microparticles in lung cancer

TMPs are capable of conferring resistance to chemotherapy. Two mechanisms are involved in MP-induced drug resistance. In the first mechanism, TMPs transport functional plasma membrane transporter proteins, including P-glycoprotein (P-gp), breast cancer resistance protein [114], and multidrug resistance (MDR)-associated protein 1 (MRP1) [115] or resistance-associated miRNAs, from drug-resistant cancer cells to drug-sensitive cancer cells [116]. MDR is innately present in tumours that arise from epithelium with a high constitutive P-gp expression [117, 118]. MDR development in cancer is clinically associated with the overexpression of the efflux transporter P-gp (P-gp, ABCB1) or MRP1 (MRP1, ABCC1) in numerous malignancies, including lung, breast, neuroblastoma, and prostate cancers [119–121]. P-gp and MRP1 belong to the ATP-binding cassette (ABC) transporter superfamily. ABC-transporters hydrolyse ATP to drive the extrusion of chemotherapeutic drugs against a concentration gradient from otherwise drug-sensitive cells. MRP1 and functional P-gp are transferred into recipient cells by MPs, imposing a donor dominant ABCC1 trait on drug-sensitive cells [116, 122, 123]. In addition to functional P-gp, MPs can also transport RNA, which can re-template recipient cells to ensure the acquisition of the donor cell MDR trait [122–124]. Some miRNAs, such as miR-27a, miR-326, and miR-451, have a potent ability to regulate ABC transporters [120, 123–126]. In the second mechanism, chemotherapeutic agents are directly expelled from cancer cells [127].

#### Microparticles and reversing drug resistance

Drug resistance remains a formidable hurdle in cancer therapy [128]. It may result from decreased drug uptake, increased drug efflux and expression of drug efflux pumps, drug inactivation/detoxification, more efficient DNA repair, and dysregulation of apoptotic pathways [129–131]. Furthermore, system cell-like cancer cells (SCLCCs) are a subset of highly tumorigenic cancer cells with the ability to self-renew and escape chemotherapy [132]. Stem cell-like tumour-repopulating cells (TRCs) play a vital role in reprogramming an immunosuppressive TME [107]. For example, TRCs cultured in vitro can replace SCLCCs and exert drug resistance. However, Ma et al. [109] showed that TMPs loaded with anti-tumour drugs can reverse the drug resistance of TRCs or SCLCCs. Delivering high concentration of drugs into soft MPs can effectively facilitate drug entry into the nucleus of tumour cells. Subsequently, soft TRCs readily undergo deformation, enabling the easy uptake of the MPs [133]. These MPs not only release drugs into the cytoplasm of TRCs, but also transport drugs into the lysosomes and nucleus, causing TRC apoptosis. Research has demonstrated objective evidence for the clinical efficacy of TMPs in patients with LC, making TMPs well tolerated in clinical practice [134].

### Immunomodulation effect of microparticles in lung cancer

Intricate interactions among the immune system, TME, and cancer cells are regulated by bioactive molecules and biological information. In human cancer cells, TMPs are more immunogenic than soluble antigens [135]. Rughetti et al. found that MP-mediated antigen transfer to dendritic cells (DCs) is crucial for the cross-presentation of tumour-glycosylated antigens [53, 54]. MP signalling strengthens the immunosuppressive properties of tumour cells, promoting the escape of immune surveillance and tumour metastasis. Moreover, MPs may trigger T cell-activated apoptosis by exposing the Fas ligand, which might contribute to immune suppression and indirectly promote tumour growth [136, 137]. However, MPs also mediate antigen presentation by exposing major histocompatibility complex class I and II molecules to DCs to facilitate immune surveillance [138]. Similarly, the lipid component of MPs can stimulate antigen presentation by activating toll-like receptor 4 on macrophages [139]. Further research indicates that the stage of tumour progression determines the conflicting effects of MPs in modulating the immune system [28].

Cancer immunotherapy makes use of innate immune response against tumours, proposing a paradigm shift in cancer therapy. The key point of this therapy is to present cancer-specific immunogens and initiate T cell-mediated cancer immunity. Due to the conflicting effects of TMPs, the relationship among cancer cells, the TME, and the immune system is complex. TMPs are generally more immunogenic than soluble antigens in both mouse models and human cancer cells [135, 140]. Mesenchymal stem cell-derived EMPs can be used to carry tumour RNA and provoke the strong anti-tumour immune response of cytotoxic CD8^+^ cells. Oral vaccination with TMPs effectively accesses and activates the mucosal epithelium, leading to anti-tumour T cell response in mouse models. The most promising therapeutic application of MPs in the field of cancer immunotherapy may be vaccines [28].

### Microparticles act as potential cancer vaccines

The fundamental principle of cancer vaccines is to provide antigen-presenting cells with both tumour antigens and immune-stimulating signals, resulting in an effective T cell immune response against tumours [141]. Zhang et al. proposed TMPs as ideal candidates for the development of novel and effective tumour vaccines [141–143]. TMPs have several applications in tumour vaccine development [144]. Apart from being potential antigen carriers, these can also directly target cancer cells. TMPs carry repertoires of tumour antigens and present these to DCs. Moreover, TMPs derived from UV-irradiated tumour cells may contain stimulatory molecules, such as DNA fragments, which stimulate DCs to produce type I interferons, interleukin (IL)-12, and interferon (IFN)-γ [145]. Type I IFNs are essential for CD8^+^ T cell priming, whereas IL-12 and IFN-γ promote antitumor T cell activation [145]. Research has shown that TMPs contain excessive immunostimulatory factors, resulting in the generation of innate immune signals in DCs [144]. Herein, TMPs contain tumor antigen spectrums and carry potential innate signals, which make them ideal candidates for developing novel therapeutic cancer vaccines. We have provided a comprehensive summary of the roles of MPs in LC patients. MPs that act as remarkable biological vectors are very promising and attractive tools for developing and exploring novel and individualised therapeutic strategies.

## Concluding remarks and future direction

**Fig. 2:**
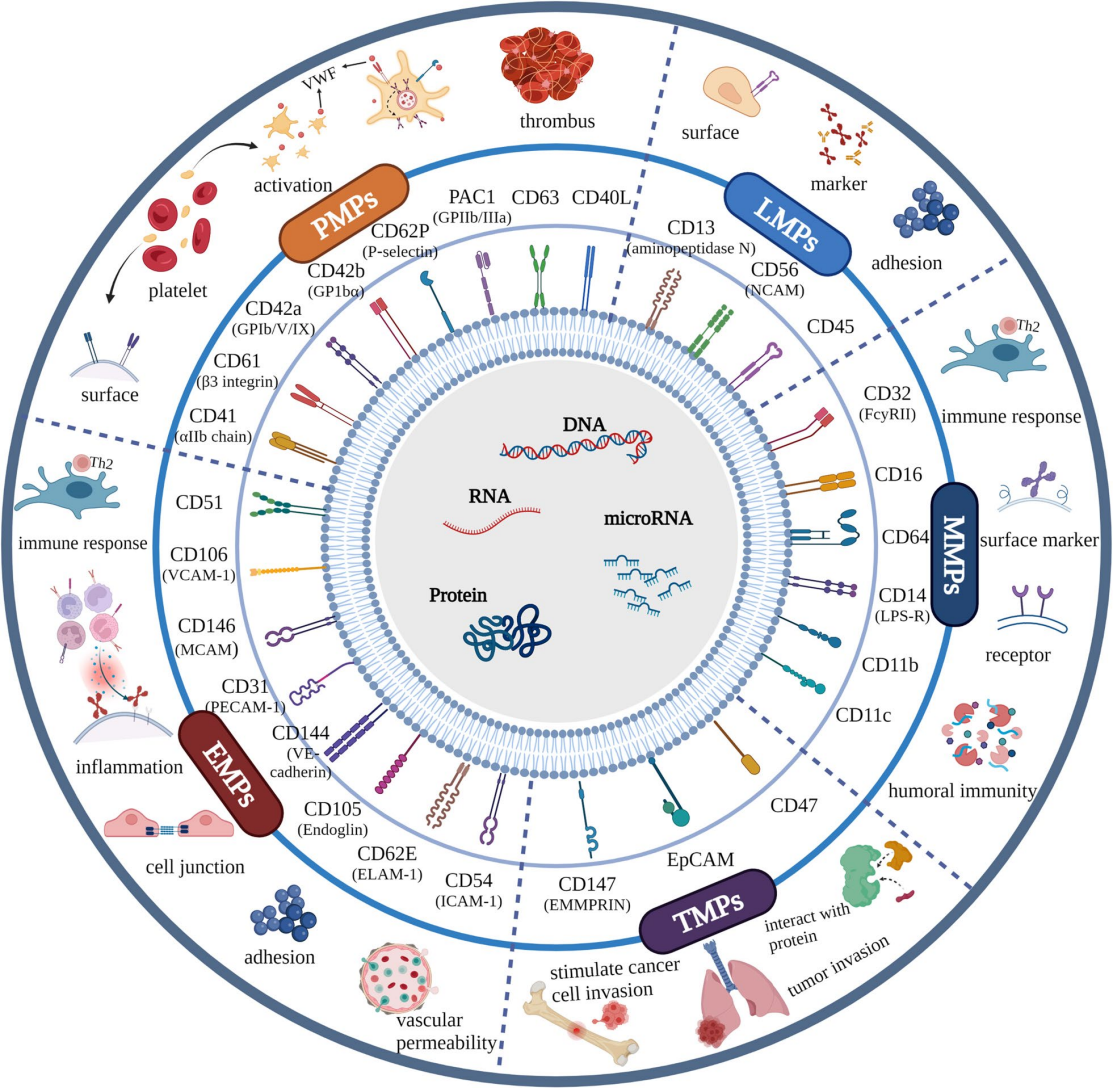
Major surface markers of different cells based on their origin and function

Numerous studies on the biology and biogenesis of MPs in cancer pathophysiology have revealed the significance of MPs in cancer growth, proliferation, apoptosis, angiogenesis, coagulation, and dissemination. In the airway and LC microenvironment, MPs derived from tumour-infiltrating cells and cancer cells are likely to play key roles in intercellular communication, promoting a microenvironment conducive to tumour growth, invasion, and metastasis (Fig. 2). Due to the evidence from current research, an increasing number of studies have suggested the possible clinical application of MPs as biomarkers. The diverse biomolecular information regarding EVs provides numerous potential biomarkers for cancer risk assessment, early detection, diagnosis, prognosis, and surveillance. To date, the development of EV-based biomarkers has largely focused on exosome biomarkers, and there are a number of key questions regarding MPs that will likely receive a great deal of research attention in the future. Several studies have investigated LC-related proteins in MPs. However, key nucleic acids are yet to be elucidated by comparing patients at different stages of LC to controls by DNA or RNA sequencing and mass spectrometry. In cancer immunotherapy, cancer vaccines are the most promising therapeutic application of MPs. Accumulated studies have investigated the involvement of MPs in lung disorders and attempted to provide new insights into the development of drug delivery systems and potential cancer vaccines. Although the exact functions and mechanisms of action of MPs have been elucidated, further research in the context of LC is necessary to ultimately develop useful means for cancer diagnosis and develop novel therapeutic strategies for various types of cancers.

## Data Availability

The data and materials that support the findings of this study are available from the corresponding author upon reasonable request.

## Abbreviations

LC: Lung cancer
EVs: Extracellular vesicles
MPs: Microparticles
EMPs: Endothelial-derived microparticles
NSCLC: Non-small cell lung cancer
TMPs: Tumour-derived microparticles
CECs: Circulating endothelial cells
IQGAP1: Ras GTPase-activating-like protein 1
BPIFA1: BPI fold-containing family A member 1
MiRNAs: MicroRNAs
PMPs: Platelet-derived MPs
EPB41L3: Erythrocyte membrane protein band 4.1-like 3
DU: Differential centrifugation
NTA: Nanoparticle tracking analysis
FCM: Flow cytometry
TRPS: Tunable resistive pulse sensing
AFM: Atomic force microscopy
DLS: Dynamic light scattering
TME: Tumour microenvironment
MMPs: Macrophage-derived MPs
LMPs: Lymphocyte-derived MPs
SPARCL1: SPARC-like protein 1
P-gp: P-glycoprotein
MDR: Multidrug resistance
MRP1: MDR-associated protein 1
ABC: ATP-binding cassette
SCLCCs: System cell-like cancer cells
TRCs: Tumour-repopulating cells
DCs: Dendritic cells
IL: Interleukin
IFN: Interferon
